# Sensitivity pattern of methicillin resistant *Staphylococcus aureus* isolated from patients admitted in a tertiary care hospital of Pakistan

**Published:** 2010-09

**Authors:** F Kaleem, J Usman, A Hassan, M Omair, A Khalid, Roz Uddin

**Affiliations:** National University of Sciences and Technology, Department of Microbiology, Army Medical College, Rawalpindi, Pakistan

**Keywords:** *Staphylococcus aureus*, MRSA, Vancomycin, Susceptibility pattern, Military Hospital

## Abstract

**Background and Objectives:**

Methicillin resistant *Staphylococcus aureus* (MRSA) is a major nosocomial pathogen causing significant morbidity and mortality. The aim of this study was to evaluate *in vitro* activities of different antibiotics against methicillin resistant *Staphylococcus aureus*.

**Materials and Method:**

The study was conducted over a period of one year (January 2009 – December 2009) in the Department of Microbiology, Army Medical College, the National University of Sciences and Technology, Pakistan. One hundred and thirty-nine Methicillin resistant *Staphylococcus aureus* isolated from the clinical specimens at Rawalpindi Military Hospital were subjected to *in vitro* susceptibility against various antimicrobials using Kirby Bauer disc diffusion technique.

**Results:**

All the isolated MRSA organisms were uniformly susceptible to vancomycin, linezolid and tigecycline. Other drugs which were found to be effective were chloramphenicol, and rifampacin. Most of the MRSA were isolated from pus samples.

**Conclusion:**

Vancomycin, tigecycline and linezolid were effective against methicillin resistant strains of *S.aureus*. This study suggests that chloramphenicol and rifampacin also have good *in vitro* efficacy for methicillin resistant *S. aureus* infections. Oral dosing option for linezolid, chloramphenicol and rifampacin can allow earlier discharge of hospitalized patients and thus reduce health care expenses as well as help reduce the chances of vancomycin resistant strains emergence.

## INTRODUCTION

The genus *Staphylococcus* includes pathogenic organisms in which *Staphylococcus aureus* is the most important. It has overcome most of the therapeutic agents that have been developed against it in the recent years (1). The introduction of beta-lactamase resistant semi-synthetic penicillins in the early 1960's provided temporary relief which ended with the emergence of Methicillin (oxacillin) Resistant *Staphylococcus aureus* (MRSA), discovered shortly after methicillin became available for clinical use (2).

Many of these MRSA isolates are becoming multidrug resistant and are susceptible only to glycopeptide antibiotics such as vancomycin. Low level resistance even to vancomycin is emerging at present (1). Prolonged hospital stays, indiscriminate use of antibiotics, lack of awareness, and receipt of antibiotics before coming to the hospital are some of the possible predisposing factors of MRSA emergence. The knowledge of MRSA prevalence and the current antimicrobial profile is necessary in selection of appropriate empirical treatment of these infections. Control of MRSA in hospitals is essential. This can be achieved by proper implementation of hospital infection control measures and regular surveillance activity. Therefore, we planned this study to determine the current antimicrobial profile of MRSA isolates from our hospital so as to formulate an appropriate as well as cost effective empirical therapy.

## MATETIALS AND METHODS

The study was conducted over a one-year (January 2009–December 2009) period at the Department of Microbiology, Army Medical College, National University of Sciences and Technology, Pakistan. All Staphylococcus aureus isolates encountered in routine clinical specimens received from clinical wards of Military Hospital Rawalpindi were identified morphologically and biochemically by standard laboratory procedures including tube coagulase test and DNase test using DNase agar (Oxoid Ltd, Basingstoke, Hampshire, England). MRSA screening was performed on Mueller-Hinton agar using 6 µg oxacillin and 30 µg cefoxitin discs as per Clinical and Laboratory Standards Institute (CLSI) guidelines (3). Susceptibility to antimicrobial agents was determined by the modified Kirby Bauer disc diffusion method using following antimicrobial discs: vancomycin (30 µg), linezolid (30 µg), teicoplanin (30µg), tigecycline (30 µg), tetracycline (30 µg), minocycline (30 µg), quinopristin/dalfopristin (15 µg), Fluoroquinolones (ciprofloxacin, levofloxacin, ofloxacin and moxifloxacin 5 µg), chloramphenicol (30 µg), rifampicin (5 µg), fusidic acid (10 µg), macrolides (erythromycin and clindamycin 15 µg) as per CLSI guidelines (3).

## RESULTS

**Fig. 1:**
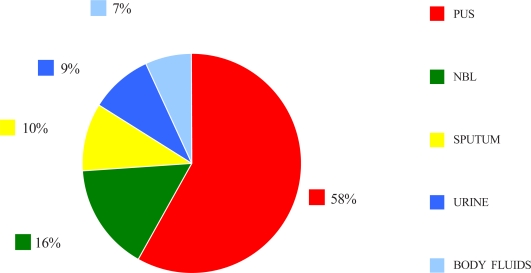
Percentage of MRSA isolated from various specimens.

A total of 139 MRSA were isolated during the study period. All of the isolated MRSA were found to be susceptible to vancomycin, linezolid and quinopristin/ dalfoprisitin. One hundred and thirty isolates (94%) were susceptible to teicoplanin and minocycline, whereas 93% of isolates were sensitive to chloramphenicol and 91% were sensitive to tigecycline. Only 38 and 22% of the isolates were susceptible to fluoroquinolones and macrolides respectively (Table 1). Majority of MRSA were isolated from pus samples followed by nasobronchial lavages samples (Fig. 1).

**Table 1:** Susceptibility pattern of MRSA against various antibiotics.

Antibiotic	Sensitive	Resistant	% sensitive
Vancomycin	139	0	100
Linezolid	139	0	100
Quinopristin/Dalfopristin	139	0	100
Teicoplanin	130	9	94
Minocycline	130	9	94
Chloramphenicol	129	10	93
Tigecycline	127	12	91
Trimethoprim/sulphamethoxazole	94	45	67
Fusidic acid	91	48	65
Tetracycline	89	50	64
Rifampicin	86	53	62
Doxycycline	57	82	41
Fluoroquinolones	53	86	38
Macrolides	31	108	22

## DISCUSSION

A study conducted at the Armed Forces Institute of Pathology Rawalpindi in 2003–2004 indicated that there was no reduced susceptibility of vancomycin against studied MRSA isolates as indicated in our study. However, VISA strains were detected in 4% of isolates in Lahore in 2004 (4, 5). The incidence of VISA in the regional countries has been documented as 3.3% (Srinagar, Kashmir, 2003), 6% (India, 2007) and 7.5% (Iran, 2008) (6–8).

The results of our study are in accordance with a study carried out at Aga Khan University in 2009 which showed variable susceptibility pattern with high resistance rates to tetracycline (82%), clindamycin (79%), cotrimoxazole (59%), and rifampicin (50%). Resistance to chloramphenicol (10%) and fusidic acid (9%) was low (9). A study carried out at Lahore in 2009 showed that only 4% of MRSA isolates were sensitive to fluoroquinoles, whereas 38% of isolates were found to be sensitive in our study (10).

In our study 100% of the isolates were sensitive to linezolid which is complemented by a 2009 study carried out in Iran. In that study, tigecycline was also found to be 100% which is similar to the results of another study in Iran (11).

Vancomycin and linezolid are highly effective against MRSA. Chloramphenicol and Minocycline also have good *in vitro* efficacy. Tigecycline, though effective, enhances health care costs enormously.

Effective antimicrobial activity as well as cost effectiveness should be considered in drugs prescribed for MRSA infections. Oral dosing options for linezolid and chloramphenicol can allow earlier discharge of hospitalized patients and minimize the chances of VRSA emergence. Good hospital infection control measures prove to be the main stay against these infections because antibiotics can never be an effective alternative to good medical practice
